# Supplementary material for: Effect of Cashew Nut Consumption on Biomarkers of Copper and Zinc Status in Adolescents with Obesity: A Randomized Controlled Trial
Source: Nutrients. 2024 Dec 31;17(1):163. doi: 10.3390/nu17010163 (PMC11723122; doi:10.3390/nu17010163)
Supplement: Supplementary file 1 [file nutrients-17-00163-s001.zip › nutrients-3385852-supplementary.pdf]

Effect of cashew nut consumption on biomarkers of copper and zinc status in adolescents with obesity: a randomized controlled trial

SUPPLEMENTARY MATERIAL

Non-Imputed Data

Table S1. Characterization of anthropometric and dietary variables in the CON and the CASN, according to intervention time.

Variables	CON (n=27)			CASN (n=54)			p-value (T0)	p-value (time and group)
	T0	T12	p-value	T0	T12	p-value		
Anthropometric								
Weight, kg	74.42 (16.52)	74.50 (16.78)	0.872	72.53 (13.65)	72.67 (13.79)	0.177	0.585	0.515
Height, m²	158.62 (10.90)	159.61 (10.80)	**<0.001**	159.94 (10.31)	161.31 (10.17)	**<0.001**	0.597	0.152
BMI, kg/m²¥	29.25 (3.79)	28.91 (3.76)	**0.048**	28.12 (2.91)	27.80 (2.82)	**0.009**	0.159	0.934
Lean mass, kg	49.42 (10.32)	48.49 (10.65)	0.277	48.58 (9.72)	48.33 (8.72)	0.474	0.781	0.637
Lean mass, %¥	70.98 (2.53)	71.06 (2.54)	0.399	71.47 (2.18)	71.48 (2.17)	0.672	0.385	0.658
Fat mass, kg	26.60 (9.60)	25.90 (8.32)	0.490	24.35 (6.37)	24.83 (7.02)	0.529	0.220	0.354
Fat mass, %	34.27 (7.59)	34.39 (5.91)	0.916	33.32 (5.84)	33.64 (4.94)	0.594	0.492	0.823
Dietetics								
Energy, kcal¥	1814.84 (1007.32)	1855.77 (885.00)	0.534	1688.70 (590.63)	1716.76 (930.71)	0.716	0.865	0.473
Protein, g¥	77.41 (40.73)	73.38 (41.95)	0.854	71.50 (27.27)	69.87 (35.60)	0.324	0.954	0.674
Protein, %¥	18.16 (7.92)	16.52 (6.12)	0.336	17.56 (5.76)	16.58 (4.96)	0.400	0.894	0.763
Carbohydrate, g¥	205.49 (106.66)	232.79 (114.46)	0.155	200.98 (75.91)	204.43 (93.81)	0.869	0.648	0.209
Carbohydrate; %	46.22 (8.93)	51.19 (12.36)	**0.049**	47.79 (8.95)	48.98 (11.39)	0.499	0.460	0.217
Lipid, g¥	64.37 (37.12)	66.89 (38.42)	0.646	64.73 (29.94)	60.36 (26.56)	0.358	0.534	0.365
Lipid, %	32.26 (7.95)	31.77 (6.89)	0.829	34.03 (8.03)	32.77 (9.79)	0.438	0.351	0.786
MUFA, g¥	19.57 (11.68)	22.15 (16.01)	0.554	20.70 (10.10)	19.74 (10.65)	0.430	0.425	0.348
PUFA, g¥	12.47 (9.90)	14.24 (9.45)	0.248	14.01 (9.30)	12.83 (7.29)	0.336	0.192	0.135
Copper, mg¥	2.90 (9.28)	1.28 (2.28)	0.662	1.25 (2.17)	0.83 (0.41)	0.085	0.804	0.518
Zinc, mg¥	10.05 (6.19)	7.48 (3.59)	0.087	8.14 (3.46)	7.36 (3.38)	0.155	0.410	0.559

Abbreviations: CASN, cashew nut group; CON, control group; T1, initial time (baseline); T12, final time; BMI, body mass index; MUFA, monounsaturated fatty acids; PUFA, polyunsaturated fatty acids. ¥: transformed variables. p-values (within-group, T0 and time and group interaction): Mixed repeated measures ANOVA corrected by Bonferroni post hoc test. p-values <0.05 were considered significant and indicated in bold.

Table S2. Characterization of CON and CASN biochemical variables, according to intervention time.

Variables	CON (n=27)			CASN (n=54)			p-value (T0)	p-value (time and group)
	T0	T12	p-value	T0	T12	p-value		
Copper, μg/dL	116.89 (19.69)	120.70 (28.35)	0.384	106.50 (22.36)	99.52 (23.66)	**0.026**	**0.044**	**0.046**
Zinc, μg/dL¥	75.89 (36.21)	148.22 (101.60)	**0.001**	65.33 (11.32)	131.30 (87.64)	**<0.001**	0.077	0.736
SOD, U/gHb	4258.32 (906.27)	4484.41 (729.58)	0.321	4492.87 (890.46)	4845.69 (907.97)	**0.030**	0.076	0.649
Ratio Cu/Zn¥	1.69 (0.42)	1.25 (0.74)	**<0.001**	1.66 (0.34)	1.11 (0.64)	**<0.001**	0.972	0.096

Abbreviations: CASN, cashew nut group; CON, control group; T1, initial time (baseline); T12, final time; SOD, superoxide dismutase; Cu, copper; Zn, Zinc. ¥: transformed variables. p-values (within-group, T0 and time and group interaction): Mixed repeated measures ANOVA corrected by Bonferroni post hoc test. p-values <0.05 were considered significant and indicated in bold.

Table S3. Nutritional status of plasma copper and zinc in the CON group and the CASN group, according to the intervention time.

Variables	T0				T12			
	All N=81	CON N=27	CASN N=54	p-value	All N=81	CON N=27	CASN N=54	p-value
Copper, n (%)								
Normal (or Low)	74 (91.4)	25 (92.3)	49 (90.7)	0.571¥	72 (88.9)	20 (74.1)	52 (96.3)	**0.005**£
High	7 (8.6)	2 (7.4)	8 (72.7)		9 (11.1)	7 (25.9)	2 (3.7)	
Zinc, n (%)								
Normal	25 (30.9)	11 (40.7)	14 (25.9)	0.174¥	54 (66.7)	21 (77.8)	33 (61.1)	0.134¥
Low	56 (61.9)	16 (59.3)	40 (74.1)		27 (33.3)	6 (22.2)	21 (38.9)	

Abbreviations: CASN, cashew nut group; CON, control group; T1, initial time (baseline); T12, final time. ¥: Pearson's chi-square test. £: Fisher's exact test.

Table S4. Variation between times (Δ T12-T0) in biochemical markers after 12 weeks of intervention in the control group (CON) and the cashew nut group (CASN).

Variables	CON<br>N=27	CASN<br>N=54	p-value
Copper, μg/dL	3.81 (15.94)	-6.98 (25.28)	**0.012£**
Zinc, μg/dL	72.33 (89.77)	65.96 (89.89)	0.109£
SOD, U/gHb	226.09 (1008.46)	352.82 (1251.06)	0.649¥
Ratio Cu/Zn	-0.44 (0.64)	-0.54 (0.69)	0.936£

Abbreviations: CASN, cashew nut group; CON, control group. ¥: Mann–Whitney test. £: Independent Student's t test.
